# The Integral Role of Organisational Governance in Promoting Interprofessional Education in Rural Settings

**DOI:** 10.3390/ijerph18063041

**Published:** 2021-03-16

**Authors:** Priya Martin, Monica Moran, Nicky Graham, Anne Hill

**Affiliations:** 1 Cunningham Centre, Darling Downs Health, Toowoomba, QLD 4350, Australia; 2 Rural Clinical School, The University of Queensland, Toowoomba, QLD 4350, Australia; 3 Western Australian Centre for Rural Health, The University of Western Australia, Geraldton, WA 6530, Australia; Monica.Moran@uwa.edu.au; 4 Speech Pathology Cairns and Hinterland Hospital and Health Service, Cairns, QLD 4870, Australia; Nicky.Graham@health.qld.gov.au; 5 School of Health and Rehabilitation Sciences, The University of Queensland, Brisbane, QLD 4072, Australia; ae.hill@uq.edu.au

**Keywords:** rural health, interprofessional education, process evaluation

## Abstract

One of the key challenges with implementing and sustaining interprofessional education initiatives is the lack of governance structures and processes to guide them. This case study presents a process evaluation of an intersectoral advisory group that facilitated a novel interprofessional clinical education model in rural health settings in the state of Queensland, Australia. The group consisted of health and academic partners to guide the implementation and promote sustainability of this new model. The advisory group process was evaluated mid-way and at conclusion of the group functions, using focus group discussions. The focus group audio recordings were transcribed verbatim and subjected to inductive content analysis. Categories were developed for reporting. Three broad categories were identified: Characteristics of the group, functions of the group and multifaceted communication within the group and between sectors. By identifying and mapping the processes used by a strategic, high-level intersectoral advisory group consisting of members from the health and academic fields, key recommendations have been formulated to guide similar work in the future.

## 1. Introduction

For over three decades, the vital role of interprofessional education (IPE) and interprofessional practice in improving outcomes for patients and health organisations has been identified and promoted internationally [1,2]. Delivery of IPE to pre-entry health professional students is a key strategy to ensure that the emerging health workforce has the required capabilities to successfully work interprofessionally, and that interprofessional practice is embedded in health service delivery in the future. It is crucial for rural healthcare settings to embed IPE in pre-registration clinical placements, as they enhance the placement experience [3], which is an important driver of rural workforce recruitment and retention [4]. 

The lack of embedded governance frameworks to support such a roll out provides significant challenges to the sustainability of IPE activities in practice settings [5,6].A growing concern is also the gap between the health and academic sectors in developing, implementing, and sustaining IPE initiatives, which can affect the sustainability of important and timely initiatives in health settings [6].

The Rural Interprofessional Education and Supervision (RIPES) model of clinical placement was developed to enhance pre-registration student clinical education capacity in rural and remote services in the state of Queensland in Australia. The RIPES model seeks to support growth in student clinical education capacity in rural and remote services, and to provide students with the opportunity to develop skills required for effective collaborative practice in health care teams [3]. The model involves multiple students from different professions undertaking clinical placement at the same site concurrently (i.e., there is a period of at least five weeks where placement dates for each student overlap). During their placement, the students participate in several structured IPE activities, in addition to their usual uniprofessional placement activities. Local student supervisors facilitate these IPE activities and are provided with training, support and relevant resources to achieve this. Each RIPES placement has two components: Regular uni-professional placement and related activities and IPE activities. The IPE activities include tutorials on competencies identified in the Canadian Interprofessional Competency Framework [1], such as interprofessional communication, collaboration, teamwork and client-centred care. Further information about the RIPES model is available elsewhere [7].

Representatives from the health and academic sectors were invited to form a high-level intersectoral advisory group to guide planning and roll out of the RIPES project. Members joined the group from varied senior work roles, including operational, strategic, teaching, research, and project management. While they drew on their expertise to support their contributions to the Advisory Group, they did not take on defined roles within the group, with the exception of the first author, who was the group secretary. The advisory group provided governance for the project, with the advisory group activities feeding into the project’s operationalisation.

Although the advisory group was convened with a set of predetermined functions, the group clarified the language/terminology used and assumptions regarding functions in the ‘forming’ phase. This led to an evolved set of functions, namely:

- To provide advice, leadership and direction for the development, implementation and evaluation of two interprofessional student placements using the RIPES model
- To facilitate successful delivery of the project through resourcing and high-level linkages and engagement with trial sites
- To monitor realisation of benefits and report to relevant stakeholders.

Whilst there is recognition that lack of governance structures and processes impede the implementation and sustainability of IPE in health settings [5,6], there is a lack of evaluation data on what these processes might look like, how effectively they may be able to support interprofessional placements in the field, and how participation impacts members. In emerging fields, process evaluation is as important as outcome evaluation. This concept is also reinforced by models such as the Tuckman’s stages of group development [8], where the group structures (i.e., processes) are acknowledged to be as important as the tasks (i.e., outcomes) the group undertakes [9]. This case study reports the process evaluation of the RIPES advisory group and is expected to fill an evidence practice gap, thus facilitating sustainability in this important area. The outcome evaluation of the RIPES model will be reported separately.

## 2. Materials and Methods

### 2.1. Setting

The RIPES advisory group meetings occurred via teleconference as members were geographically dispersed across two states. Meetings were facilitated by the chair of the advisory group. An agenda was distributed to members via email prior to each meeting, along with minutes and an action plan from the previous meeting. The advisory group held a total of 11 meetings between January 2017 and July 2018.

### 2.2. Participants

The RIPES advisory group consisted of nine members from the health and academic sectors who attended the regular group meetings and discussions. Two members were IPE experts from universities in Queensland and Western Australia. Of other members, some held state-wide roles in the Queensland public health sector, and others were representatives from the two project implementation sites. The group included members working in junior and senior roles and representing a breadth of roles, including strategic, managerial, operational and research. All members had the opportunity to contribute to the process evaluation. Those wishing to be excluded from the process evaluation were free to do so.

### 2.3. Data Collection

Focus group discussions were facilitated by the first and second authors with participants via teleconference at the halfway point (*n* = 8; October 2017) and after the final advisory group meeting (*n* = 7; July 2018). Members who were unable to attend the focus groups had the opportunity to provide input via email, with one group member taking up this option. The discussions were recorded with permission, and 67 min of the recording were transcribed verbatim by an independent typist. A focus group guide with trigger questions and prompts was developed to guide both focus groups. The guide has been attached as Appendix A.

### 2.4. Data Analysis

The focus group data and email transcripts were analyzed by the first two authors using an inductive content analysis approach. Within this approach, the transcripts are read and re-read, and concepts developed from the data [10]. Accordingly, the first two authors read the transcripts several times and developed categories independently. Subsequently, they met to discuss the categories until consensus was reached [10]. To enhance the trustworthiness of the analysis process, the third and fourth authors also reviewed the data and validated the categories. The final categories were the products of consensus reached during the discussion with all authors. Moreover, the focus group findings were shared with the group members for their comments and feedback as an added strategy to further enhance the trustworthiness of the findings. Our decision to use this qualitative evaluative approach (i.e., collecting data through focus groups and applying content analysis to the data) was driven by a need to understand complex processes surrounding IPE advisory group formation and implementation.

### 2.5. Ethics Approval

The project proposal was considered by the Darling Downs Health Human Research Ethics Committee for multi-sites, which provided an exemption to a full review on the basis of it being a service evaluation and not research (HREC/17/QTDD/62; dated 13 July 2017).

## 3. Results

Three broad categories were developed following the inductive content analysis process.

### 3.1. Characteristics of the Group

Group members noted that the advisory group was very committed to facilitating project outcomes. Members, although from different backgrounds, such as strategic, operational, educational, project management and managerial roles, functioned collaboratively without competing with each other. Members further noted that all were respectful of each other and, although from different professional backgrounds, were able to work collaboratively in an interprofessional manner. One member observed that the group formation phase was enabled by the fact that several members had already known each other in a different capacity.

Whilst reflecting on the group process, one member said thus:

… there is not a lot of ego or uni-professional competition in the group … people have really focused just on the project and are working together and not worried about some of those things that sometimes in the workplace cause roadblocks

Another member commented thus:

… I thought it (the group) felt very collaborative, equal, respectful and also everyone committed to doing the task they were asked to do

Whilst reflecting on the skills mix in the group, another member commented:

I think we had a really good mix of expertise within the group...some that had really great expertise in evaluation and tools for assessing interprofessional practice and experience in project management and linkage with other health staff...I think we had a really good blend across the group …

Comments were also made about different members taking on a leadership role at different points in time and leveraging off different skill sets depending on what was required. Whilst commenting on this, one member noted:

… this group knows roughly who might be in a position to provide advice on specific matters and so the meeting, I find, tends to swing to that individual that has the expertise or works for a particular work unit, who is likely to have a stake or particular responsibility for providing advice on a particular issue.

### 3.2. Functions of the Group

There was agreement that the group created linkages, shared resources and provided professional development opportunities for members. One member, when asked about the impact of the advisory group on members’ own interprofessional competencies, said thus:

… I think my knowledge has increased exponentially. I guess it was nice going to the (IPE) workshop on Friday and thinking that I actually kind of know a bit about this stuff and can contribute. From a personal perspective, (the project has impacted on my IP competencies) very much

Another member, when describing the collaborative leadership enacted within the group, said:

… I feel like my role has been very clear to me and it sounds like that was the case with others as well. I think the other thing is collaborative leadership that [x] just mentioned … It’s nice to be involved in a group that further develops that collaborative leadership

### 3.3. Multifaceted Communication

Members observed key features of communication used within the group and between sectors and sites, whilst also noting the challenges of finding time and coordinating meeting time.

It is noteworthy that members reflected more about the ‘forming’ phase at the second focus group and agreed that the advisory group could have functioned more efficiently during its formation if the roles and expectations of the group members had been communicated more explicitly. Whilst reflecting on the barriers, one member said:

… even very early on in the formation of the group, trying to work out what our roles were. Was it an advisory group or was it a steering committee … what was the [coordinating organisation] wanting us to do?

Some complexities around the role clarity of the group and of individual members within the group were also noted at the second focus group. This, in combination with the need to have a shared language, was, in turn, attributed to the complex nature and interpretation of the concept of IPE. One member said:

… the first six months or so, I think one of the fundamental challenges with this was IPE itself. You know, you say the word and I think of a giraffe and the next person thinks of a rhinoceros and the next person thinks of a hyena...thinking back on, you know, many, many drafts trying to work out what the project was about…

Some members of the group had worked together previously and were aware of each other’s roles. This was noted to be an enabler as not having this pre-existing knowledge could have meant the group spent more time in the ‘forming’ phase to get to know each other and understand others’ roles. One member commented thus:

… There is an element of distributed leadership within the group, but I think with an appreciation that most of us within the group know each other quite well, and…understand where the expertise within the group’s members lie … If we had come in with no pre-existing knowledge about other members … the project would have required a longer ‘warming’ phase …

One member stressed the importance of keeping up with two-way communication throughout the duration of the group’s existence. She said:

… I sort of feel like we had a lot of flurry of input and involvement at the start but then once the placement was happening, it kind of stopped.

As members were from two states in Australia and, therefore, in different time zones as well as from different organisations, difficulties in scheduling meetings, as well as finding time for meetings whilst in busy roles, were noted by several members. However, the group also noted that these barriers were overcome because of the commitment members had. One member said:

… even though we don’t always have everyone able to link in, we have had members sending in things via emails … we are well communicated to … We get information from those people who haven’t been able to attend, which I think is great. So, the commitment is still great.

## 4. Discussion

This study evaluated the processes and perspectives of the RIPES advisory group in facilitating the establishment, implementation and evaluation of an interprofessional student placement project across two rural work locations. Analysis of focus group data revealed three categories related to group characteristics, group function, and multifaceted communication. Results highlight the enablers and challenges, lessons learned and recommendations that can be used as a roadmap for success in building sustainable processes for intersectoral leadership groups in order to maximise interprofessional project outcomes in rural settings.

Group members identified several enablers which supported the group’s ability to exert leadership in order to scaffold the development of the program. The commitment of members was considered key to group success. The notion of collaborative or distributed leadership amongst the interprofessional group was considered a strength of the group and enabled sharing of skill sets and resources. Collaborative leadership is recognised as a common model of leadership in IP teams and has been reported to facilitate effective team processes [1]. Collaborative leadership in the current project was enabled through members demonstrating mutual respect, having a good mix of expertise, having had prior working relationships and not being profession-centric. Effective communication, which was multifaceted and enabled a shared language, also maximised collaborative practice and subsequent team processes [1,11].

While research on knowledge diversity and team performance has stressed the benefits and value of diversity in teams [12], group members reflected on the challenges that this created. Language differences around the definition of IPE initially hampered group communication. Significant time investment was required to establish a shared mental model of group purpose, roles, and responsibilities as well as a shared understanding of where knowledge was located among team members. Although this ‘forming’ phase, common to every small group [8], is expected to take time, groups focused on IPE may benefit from additional time to initially work through what IPE is and is not. Scheduling suitable meeting times around different organisations, geographic locations and time zones was an operational challenge and required flexibility to enable all group members to contribute in some way to all meetings.

We have learnt that establishing the right advisory group for the development, implementation and evaluation of an interprofessional student program in rural settings is time well spent. Tapping into committed senior leaders with experience of program development and knowledge of IPE competencies and activities accelerates decision making, provides evidence to support actions and creates synergies between various stakeholders. When building an advisory group, we recommend stakeholders consider the group work and context and be clear on who needs to be at the table, potentially crossing typical organisation boundaries to ensure the full skill set required to deliver on the project. Allowing sufficient time at the start of the project to clarify roles, identify resources within the group, agreeing on terms of reference and shared language is also critical to group efficiency and success. Should the group members not have pre-existing working relationships and information about each other’s roles, further time needs to be allowed in the initial ‘forming’ phase to account for this. Furthermore, flexibility in communication methods (i.e., having more than one option, including telephone, email) assists in managing scheduling challenges and ensures all team members can continue to contribute to tasks throughout the project lifecycle.

Based on the process evaluation findings, we also offer the following recommendations to project managers and other interested stakeholders. This study found that group member satisfaction in the group process and functions was linked to both overall project outcomes and individual members’ perceived benefits. As such, participation in the advisory group should be viewed as not only a contribution to project outcomes but also as valuable to participants’ own professional development as they develop knowledge and skills that would support interprofessional practice and future collaborations. Grounding group processes and functions within models of interprofessional collaborative practice and collaborative leadership [13] and small group development [8], will maximise contributions by all members. Lessons learned from this evaluation have yielded recommendations that have enhanced project sustainability.

Many new practice-based IPE projects fade away as they are dependent on the commitment of local champions without higher-level organisational support across the health and education sectors. The RIPES project is now in its second phase, and while its success is due to a range of elements, the support and influence of the advisory group are major contributing factors to its sustainability. Conducting a process evaluation of governance and advisory groups provides greater clarity and transparency around the value and benefits they can add to promote project success and sustainability. Lessons learnt from this process evaluation have also been useful in informing the setting up of other state-wide IPE projects in Queensland, such as the IPE toolkit and interprofessional practice framework projects.

Whilst this evaluation explored the perspectives of an interprofessional senior leadership group with members situated in multiple sites, it may be limited in its application to other contexts, professions and projects. Further research could determine whether advisory group characteristics and functions are similar in larger groups of different professions and in a variety of projects. In addition, it would be important to investigate the sustainability of the group’s function over longer terms and where significant organisational change is a focus.

In addition to the evaluation of the advisory group process, the RIPES project was also evaluated to determine the project outcomes. Evaluation findings indicated that the RIPES model had perceived benefits to the students on placement, their clinical educators and the organisational work units that hosted these placements. Following the evaluation findings, the RIPES model was refined and a further phase of the project (RIPES phase two) initiated. This phase involves the implementation of the RIPES model of placement in a further five sites in Queensland and rigorous evaluation as a multi-site, multi-methods research study. Three members from the RIPES advisory group are involved in phase two to ensure project continuity, demonstrating the sustainability of the advisory group processes. Project outcomes from this phase will be reported separately as it becomes available.

## 5. Conclusions

Based on our study findings, we offer the following recommendations: IPE advisory groups need to cross intersectoral boundaries to ensure the right skill mix and expertise is available. More time needs to be allowed in the ‘forming’ phase to ensure members develop a shared understanding of IPE terminology and concepts, as well as understand their roles and expectations. Additional time needs to be factored in the ‘forming’ phase if group members had little to no pre-existing working relationships or knowledge about each other’s roles. Different ways of communication need to be made available, such as videoconference, telephone and email. Meeting dates and times need to alternate to accommodate availability of members in different sectors or time zones. Group membership needs to consist of those with expertise in IPE evaluation, project management, administration, strategic oversight and include representation from project implementation sites. Using a model such as the Tuckman’s stages of group development to ensure smooth transitioning of the group stages as work progresses. These strategies will ensure an advisory group that has the right mix of skills, expertise and influence, and also ensure that the project is fit for purpose, contextual and sustainable.

The formation and operationalisation of the RIPES intersectoral advisory group provided a range of vital support functions for the success of the project as well as developmental opportunities for members of the group. The support and governance function provided a dynamic link between senior management and the personnel delivering the project on the ground. A well-identified risk to the sustainability of IPE activities in practice settings is a lack of such intersectoral support. This case study provides a model for embedding an interprofessional and intersectoral governance framework to support the development and sustainability of novel interprofessional activities.

## Data Availability

Data and materials are protected by ethics but may be made available in de-identified format upon contact made to the corresponding author.

## Appendix A

**Focus Group Guide with trigger questions**

- What is the role/s of the advisory group in facilitating RIPES placements/organisational change?
- What strategies have we used so far to achieve project goals?
- What barriers have we experienced so far while trying to fulfil our roles on the advisory group?
- What are the perceived successes to date in terms of members’ contribution to the project?
- What are the perceived challenges to date in terms of members’ contribution to the project?
- Comment on your satisfaction as a group member with your role and project outcomes to date.
- What changes, if any, does the group need to make to ensure project outcomes are met effectively and within stipulated timeframes?
- How have leadership functions been enacted in the group?
- Thinking about interprofessional competencies (e.g. client-centredness, interprofessional communication, role clarification, team functioning and conflict management)
  – Has working on this project impacted on your competency in any of these areas? Please describe.
  – Which competencies have been particularly required for working on this project? Please think of examples from your experience.
